# The Effect of Seasonal Ambient Temperatures on Fire-Stimulated Germination of Species with Physiological Dormancy: A Case Study Using *Boronia* (Rutaceae)

**DOI:** 10.1371/journal.pone.0156142

**Published:** 2016-05-24

**Authors:** Berin D. E. Mackenzie, Tony D. Auld, David A. Keith, Francis K. C. Hui, Mark K. J. Ooi

**Affiliations:** 1 Centre for Ecosystem Science, University of New South Wales, Kensington, NSW, 2052, Australia; 2 Ecosystem Management Science, Office of Environment and Heritage (NSW), PO Box 1967, Hurstville, NSW, 2220, Australia; 3 Mathematical Sciences Institute, Australian National University, Canberra, ACT, 2601, Australia; 4 Institute for Conservation Biology, University of Wollongong, Wollongong, NSW, 2522, Australia; Wuhan Botanical Garden, Chinese Academy of Sciences, CHINA

## Abstract

Dormancy and germination requirements determine the timing and magnitude of seedling emergence, with important consequences for seedling survival and growth. Physiological dormancy is the most widespread form of dormancy in flowering plants, yet the seed ecology of species with this dormancy type is poorly understood in fire-prone vegetation. The role of seasonal temperatures as germination cues in these habitats is often overlooked due to a focus on direct fire cues such as heat shock and smoke, and little is known about the combined effects of multiple fire-related cues and environmental cues as these are seldom assessed in combination. We aimed to improve understanding of the germination requirements of species with physiological dormancy in fire-prone floras by investigating germination responses across members of the Rutaceae from south eastern Australia. We used a fully factorial experimental design to quantify the individual and combined effects of heat shock, smoke and seasonal ambient temperatures on germination of freshly dispersed seeds of seven species of *Boronia*, a large and difficult-to-germinate genus. Germination syndromes were highly variable but correlated with broad patterns in seed morphology and phylogenetic relationships between species. Seasonal temperatures influenced the rate and/or magnitude of germination responses in six species, and interacted with fire cues in complex ways. The combined effects of heat shock and smoke ranged from neutral to additive, synergistic, unitive or negative and varied with species, seasonal temperatures and duration of incubation. These responses could not be reliably predicted from the effect of the application of single cues. Based on these findings, fire season and fire intensity are predicted to affect both the magnitude and timing of seedling emergence in wild populations of species with physiological dormancy, with important implications for current fire management practices and for population persistence under climate change.

## Introduction

Many plant species in fire-prone regions worldwide rely on recruitment from soil seed banks for population persistence in the face of recurring fires [1–3]. Developing a mechanistic understanding of the processes that govern this critical life history stage is essential to our ability to predict and manage species’ responses to environmental change [4]. The first step is to characterise species’ dormancy and germination requirements as these control the timing of seedling emergence [5] which has important consequences for seedling survival and growth [6–9], and flow on effects for population dynamics and community composition [10].

Physiological dormancy (hereafter referred to as PD) is the most common and phylogenetically widespread form of dormancy in angiosperms, and is overcome by various processes such as stratification and after-ripening [11]. Once dormancy is broken, non-dormant seeds persist in the seed bank and remain viable but delay germination until they receive certain cues [12]. A specific seasonal temperature is the most common primary cue and this ensures that seedling emergence coincides with the time of year when conditions are most favourable for successful establishment [5]. In fire-prone ecosystems, however, seeds with PD may require additional fire-related stimuli such as heat shock and/or smoke to promote significant levels of germination [13–16], resulting in a seasonal pulse of post-fire seedling emergence [17,18].

Given that fire is a frequent and predictable occurrence in many of the world’s major biomes [19], and that PD is so widespread within these vegetation types [11,16,20], surprisingly little is known about the seed ecology of species with PD in fire-prone regions [21]. This is attributable to several factors. Firstly, the role of season and seasonal temperatures in the seed ecology of fire-prone floras has often been overlooked due to a focus on fire as the driving force of recruitment in these systems [15]. This focus is consistent with observations of extensive seedling emergence confined to the first or second year post-fire [22–25]. In addition, many fire-prone regions, including the Mediterranean Basin, southern California, south western Australia and the western Cape region of South Africa, have Mediterranean-type climates characterised by hot, dry summers and cool, wet winters. Highly seasonal rainfall restricts post-fire germination and seedling to emergence to the wetter months, providing less impetus to investigate seasonal temperatures as key drivers of germination in these regional floras. A number of other fire-prone regions, however, including the eastern Cape of South Africa, eastern USA and south eastern Australia, have non-Mediterranean climates with aseasonal rainfall patterns. This means that germination can potentially occur at any time of year, and that seasonal germination cues—not only direct fire cues—could play an important role in seedling emergence patterns in these regions [15].

A further factor hampering our understanding of PD species and fire is a lack of data regarding the combined effects of multiple fire-related cues and environmental cues on their germination. Considerable work conducted in fire-prone regions has demonstrated that germination of many species, predominantly those with physical dormancy, is promoted by heat shock [23,24,26–31], while many other species respond to smoke [29,32–36]. The combined effect of heat shock and smoke on germination, however, is less often assessed than the individual effects of these cues. Reports of the combined effect of heat shock plus smoke range from neutral to additive [37–39], synergistic [38,40,41], unitive (both cues are required for a response) [40,42,43] and negative (e.g. heat shock diminishing the smoke response) [15,33]. There is additional complexity due to the possibility of interactions between fire cues and environmental cues such as light [40,44], seasonal temperature [13,15,17,45] and moisture [46]. Responses to single cues tested in isolation under laboratory conditions, especially null responses, may, therefore, be unreliable indicators of germination responses under field conditions where numerous, potentially interacting cues coincide. Hence, factorial assessments of multiple putative cues are needed to draw accurate conclusions about their effectiveness and to advance our understanding of seed ecology and species with PD in fire prone regions.

Fire season is a relatively understudied component of the fire regime with the potential to impact on recruitment patterns in a diverse range of species [47]. Significant delays in post-fire seedling emergence can occur in species which require both a seasonal cue and a fire cue, as germination is restricted to a particular season while the season of fire can vary [18,21]. The magnitude of recruitment may also be affected [21]. The majority of research into fire season impacts on recruitment has focused largely on non-dormant, serotinous taxa [48–52]. Seminal papers by Le Maitre [53] and de Lange and Boucher [13] highlighted the potential impacts of fires in different seasons on PD species with soil-stored seeds but there has been a paucity of subsequent research. A field study in the highly seasonal rainfall climate of south western Australia by Roche *et al*. [17] found that application of aerosol smoke in different seasons, as an analogue of fire passage, significantly impacted the timing and magnitude of emergence and subsequent seedling survival for a variety of species with forms of PD. The inferred mechanism was a requirement for seasonal germination temperatures. In the aseasonal rainfall region of south eastern Australia, Ooi *et al*. [15,18] documented delayed emergence in relation to fire season in *Leucopogon* spp. (Ericaceae), and demonstrated a mechanistic link with seasonal germination requirements. A follow-up study by Ooi [9] found that late-emerging seedlings had experienced higher mortality and suppressed growth and maturation rates as a consequence of seasonally-imposed delays in emergence. For the vast majority of species, however, the impacts of fires in different seasons are unknown.

Prescribed burning for bushfire hazard reduction is already causing regional shifts in peak fire season [54]. While the effects of such fires may be relatively localised, their impact on regionally significant, locally endemic, or threatened species could be considerable. Furthermore, climate projections for much of southern Australia are for warming and drying with an increase in extreme fire-weather days, and a concomitant increase in fire frequency and intensity [55]. The main season for wildfires is also predicted to widen [56]. Moreover, climate change is expected to have widespread impacts on seed bank dynamics and emergence patterns via its influence on ambient temperature and soil moisture, both key drivers of seed dormancy and germination [10]. Hence, there is an urgent need to develop a mechanistic understanding of how fire and seasonal cues interact and affect seed bank dynamics and recruitment in species with PD.

The Rutaceae is an important cosmopolitan family and is one of the most significant plant families in the Sydney region of south eastern Australia, comprising a large proportion of rare and nationally threatened species [57]. Rutaceae are widely reported as difficult to germinate [34,58], however, rarely have cues that mimic those occurring in natural populations been applied to viable, non-dormant seeds [57]. Very few studies have assessed multiple germination cues in combination with one another, and none that we are aware of have done so in conjunction with a full a complement of seasonal temperatures. As a result, our understanding of the impact of fire on the seed ecology of Australian Rutaceae is poor and this is limiting effective management of the family in fire-prone habitats [57].

## Aims

We aimed to improve understanding of the seed ecology of Australian Rutaceae, and of fire-prone species with PD more generally, by investigating the role of seasonal ambient temperatures and fire-related cues in the germination of the largest Australia genus, *Boronia*. The approach we take recognises that, for many species, a germination event comprises two distinct phases: dormancy alleviation and the subsequent stimulation of germination [11,16,59–61]. An important ecological distinction is thus maintained between mechanisms that overcome dormancy, which is a characteristic of the seed, and environmental cues that subsequently promote germination once dormancy has been broken [11,16,59–61]. We focused on stimulating germination of fresh seeds by mimicking environmental cues that seeds would be exposed to naturally *in situ*. Specifically, we asked:

1. Do fire-related cues such as heat shock and smoke promote germination, and if so, how do they interact with each other?
2. Is there an effect of seasonal ambient temperatures on the germination responses to fire-related cues?
3. How variable are germination syndromes within the genus, and can patterns be explained by differences in seed morphology or phylogenetic relatedness?
4. What are the implications of these germination syndromes for post-fire emergence patterns and current fire management practices?

## Methods

### Study area, study species and seed collection

The study was conducted in the Sydney region of south eastern Australia, with the approval of the New South Wales Office of Environment and Heritage (Scientific Licence No. SL101105). The regional climate is temperate with no dry season, according to the Köppen classification [62]. As rainfall is distributed relatively evenly year-round, germination can potentially occur at any time of year *cf*. Mediterranean-type climates where dry summers usually restrict germination to the cooler, wetter months.

We selected study species based on their fecundity, comparable flowering phenology, and our ability to locate large enough populations to gather sufficient seed. The resulting seven species comprise a mixture of rare and common shrubs from fire-prone heaths and woodlands, and represent three different evolutionary lineages within the genus (Table 1). Mature seeds were collected at the time of natural seed dispersal using light-weight, polypropylene bags. Bags were tied around fruiting branches prior to seed release as initial dispersal of *Boronia* seeds is ballistic [63]. Two distinct polymorphs (*viz*. black seeds and brown seeds) were evident in the *B*. *anemonifolia* subsp. *anemonifolia* (hereafter *B*. *anemonifolia*) collection, however, the latter were discarded as they were typically (74 ± 6.2%, *n* = 50) unfilled with low viability (7.7 ± 7.4%) of filled seeds. Different maturation times between species and logistical constraints meant that the time elapsed between seed collection and commencement of experiments ranged from 5 weeks (*B*. *anemonifolia*, *B*. *fraseri*, *B*. *pinnata*, *B*. *serrulata*) to 9 weeks (*B*. *floribunda*, *B*. *ledifolia*, *B*. *thujona*), during which seeds were stored in envelopes under ambient temperatures varying between c. 20–25°C. Seed weights were determined using an electronic balance accurate to 0.00001 grams. Seed fill and viability were assessed via a cut test [64], whereby dissected seeds with firm, moist, white endosperm and healthy-looking embryos were scored as viable. Seed coats were assumed to be water-permeable as physical dormancy (hard-seededness) is absent in the family [58].

**Table 1:** *Boronia* species and seed collection sites used in this study. All sites are within the Sydney region of south eastern Australia.

Species	Section [65]	Habitat [66]	Regional Significance [66]	Study Site	Latitude, Longitude
*B*. *anemonifolia* subsp. *anemonifolia*	Cyanothamnus	Among rocks in open forest and heath	Widespread on coast and ranges	Evans Lookout, Blue Mountains National Park	-33.648993, 150.327005
*B*. *floribunda*	Boronia	Ridgetops and rock outcrops in open forest and heath	Local endemic	Scouters Mountain, Heathcote National Park	-34.069024, 150.994857
*B*. *fraseri*	Valvatae	Gullies in moist eucalypt open forest	Rare local endemic	Campfire Creek, Blue Mountains National Park	-33.783740, 150.593632
*B*. *ledifolia*	Valvatae	Ridges and rocky outcrops in woodland	Widespread on coast and ranges	Warumbul Road, Royal National Park	-34.084327, 151.072441
*B*. *pinnata*	Boronia	Ridges and plateaus in eucalypt forest and heath	Chiefly coastal	Narrowneck Peninsula, Blue Mountains National Park	-33.735867, 150.282776
*B*. *serrulata*	Boronia	Rock outcrops and platforms in moist heath and woodland	Rare local endemic	Scouters Mountain, Heathcote National Park	-34.085657, 150.987191
*B*. *thujona*	Boronia	Gullies, creeks, clifflines in moist eucalypt open forest	Northern limit of distribution	Flat Rock Creek, Royal National Park	-34.113877, 151.066522

### Experimental design

The individual and combined effects of heat shock, smoke and seasonal ambient temperatures on the germination of fresh seed were tested in a fully orthogonal laboratory experiment with 12 possible factorial combinations of heat shock (2 levels: heated, unheated), smoke (2 levels: smoked, unsmoked), and seasonal temperature (3 levels: representing summer, autumn/spring, and winter). Seed availability varied between species and enabled replication within treatments as follows: 4 replicates of 25 seeds each for *B*. *ledifolia*; 4 replicates of 20 seeds each for *B*. *anemonifolia*, *B*. *floribunda*, *B*. *fraseri* and *B*. *serrulata*; 4 replicates of 18 seeds each for *B*. *pinnata*, and 3 replicates of 24 seeds each for *B*. *thujona*. Heat shock treatments were applied by placing seeds in aluminium foil cups and exposing them to 90°C in a pre-heated oven for 10 minutes. This temperature and duration of heating falls within the range of conditions experienced by seeds in the upper layers of the soil during the passage of fire [67], and has been reported to promote germination of a wide range of local species [27,68], including several Rutaceae [69]. Smoke treatments were applied by placing seeds in aluminium foil cups inside a 60 L plastic chamber and piping in aerosol smoke for 10 minutes. Smoke was generated using a bee smoker to combust fresh leaves, dry litter and fine fuels collected from sclerophyll woodland. This method and duration of smoke exposure is reported to enhance germination of a number of species in the region [39,42,69]. Replicate heat shock and smoke treatments, respectively, were applied independently to avoid pseudoreplication [70], and heat shock treatments were applied first in the case of combined heat shock and smoke treatments.

Replicates of seeds were placed on moistened filter paper in 9 cm Petri dishes. Dishes were sealed with plastic wrap to reduce desiccation and then placed inside temperature- and light-controlled incubators set to one of three seasonal temperature regimes (Table 2) on a 12h/12h light/dark and maximum/minimum temperature cycle. The temperature inside each incubator was monitored at 90 minute intervals throughout the trial using temperature loggers (iButton Thermochron 1921G) to ensure target temperatures were being reached. The position of dishes within each incubator was rotated weekly to minimise the chance of any position effect. Filter paper was kept moist by watering with distilled water as required. Germination was scored weekly on emergence of the radicle for 14 weeks with the exception of weeks 8, 11 and 12 in which trays went uncensused. Incubation for longer periods than this would not have been ecologically meaningful as seeds do not experience the same seasonal temperature regimes under field conditions for more than three months at a time. Ungerminated seeds were assessed for fill and viability post-trial.

**Table 2:** Seasonal incubation temperatures derived for each of the study species using long-term datasets from nearby weather stations [71]. Refer to Table 1 for locations of seed collection sites.

Species	Summer incubation temp. (°C)	Autumn/ spring incubation temp. (°C)	Winter incubation temp. (°C)	Nearest weather station to seed collection site	Mean summer max/min temp. (°C)	Mean autumn/ spring max/min temp. (°C)	Mean winter max/min temp. (°C)
*B*. *floribunda*	26/17	22/12	16/7	Lucas Heights	26/17	22/12	16/7
*B*. *ledifolia*							
*B*. *serrulata*							
*B*. *thujona*							
*B*. *fraseri*	26/17	22/12	16/7	Springwood*	27/16	22/12	16/7
*B*. *anemonifolia*	22/12	16/7	10/3	Mount Boyce	23/13	17/8	10/3
*B*. *pinnata*							

*The nearest weather station to the *B*. *fraseri* site is actually Glenbrook but it does not provide temperature data.

### Data analysis

Analyses were conducted using R 3.2.2 [72]. The number of seeds germinated out of the number of filled seeds in each petri dish was assumed to follow a binomial distribution with probability *π* and was analysed using three-factor, orthogonal generalised linear models (GLMs) with logistic link functions. Models took the form *η* = *β*_0_ + *β*_1_ × H + *β*_2_ × S + *β*_3_ × Se_Aut_ + *β*_4_ × Se_Win_ + *β*_5_ × H · S + *β*_6_ × H · Se_Aut_ + *β*_7_ × H · Se_Win_ + *β*_8_ × S · Se_Aut_ + *β*_9_ × S · Se_Win_ + *β*_10_ × H · S · Se_Aut_ + *β*_11_ × H · S · Se_Win_ where *η* is the logit proportion of filled seeds germinated, *H* is the effect of heat shock, *S* is the effect of smoke treatment, *Se*_*Aut*_ is the effect of autumn/spring temperature treatment, *Se*_*Win*_ is the effect of winter temperature treatment, and *β*_0_-*β*_11_ are regression coefficients with *β*_0_ corresponding to the control treatment at summer temperatures. The proportion of filled seeds germinated is given by *π* = *e*^*η*^ / (1 + *e*^*η*^).

Models were fitted to the data after six weeks and again after 14 weeks at the conclusion of the experiment. We used the maximum germination response observed across all treatments after six weeks to estimate the proportion of seeds that were non-dormant at dispersal (any seeds that germinate must, by definition, be non-dormant, and, within the context of PD species and our research approach, heat shock and smoke do not break dormancy; they stimulate germination of non-dormant seeds [14,16,60,73]). We arbitrarily chose six weeks’ incubation as a cut-off for this estimate, given that extended periods of stratification have the potential to overcome PD and promote greater germination, and we assumed that the effect of this duration of incubation on primary dormancy had been minimal. The germination responses observed after 14 weeks provided a measure of the effectiveness of prolonged stratification under laboratory conditions in alleviating dormancy.

Model parameters were estimated using Bayesian Markov Chain Monte Carlo (MCMC) methods with uninformative priors. Sampling was performed in JAGS [74] via the package ‘R2jags’ [75]. Three chains, each comprising 100,000 iterations, were used in the MCMC process with a burn-in of 50,000 iterations and a thinning rate of 10, giving a combined total of 15,000 samples for each posterior distribution. Models were considered to have converged when traceplots were well mixed and the Gelman-Rubin statistics were below 1.1. Model adequacy was assessed using graphical posterior predictive checks and Bayesian *p*-values [76]. Bayesian 95% credible intervals for all parameters and contrasts were estimated from the posterior distribution. Temporal patterns in germination were modelled using non-parametric Kaplan-Meier estimates of survivor functions computed by the R function survfit from the ‘survival’ package [77].

Mixing of chains was good except in several datasets with a high incidence of extreme probabilities (i.e. 0% germination in some treatments). In such cases, placing an arbitrary constraint on the linear predictor (i.e. [–100, 100]) substantially improved chain convergence [76]. For *B*. *thujona* after 6 weeks’ incubation however, the 3-way interactions between heat shock, smoke and seasonal temperatures had to be dropped from the model to enable convergence. Overdispersion detected in the GLMs for *B*. *thujona* after 6 weeks and after 14 weeks, respectively, and in the GLMs for *B*. *floribunda*, *B*. *pinnata* and *B*. *serrulata* after 14 weeks, was effectively addressed by fitting generalised linear mixed models (GLMMs) with an observation-level random effect for Petri Dish, *ε*, where *ε ~ N*(0,*σ*^2^).

## Results

### Seed traits and primary dormancy

**Fig 1:**
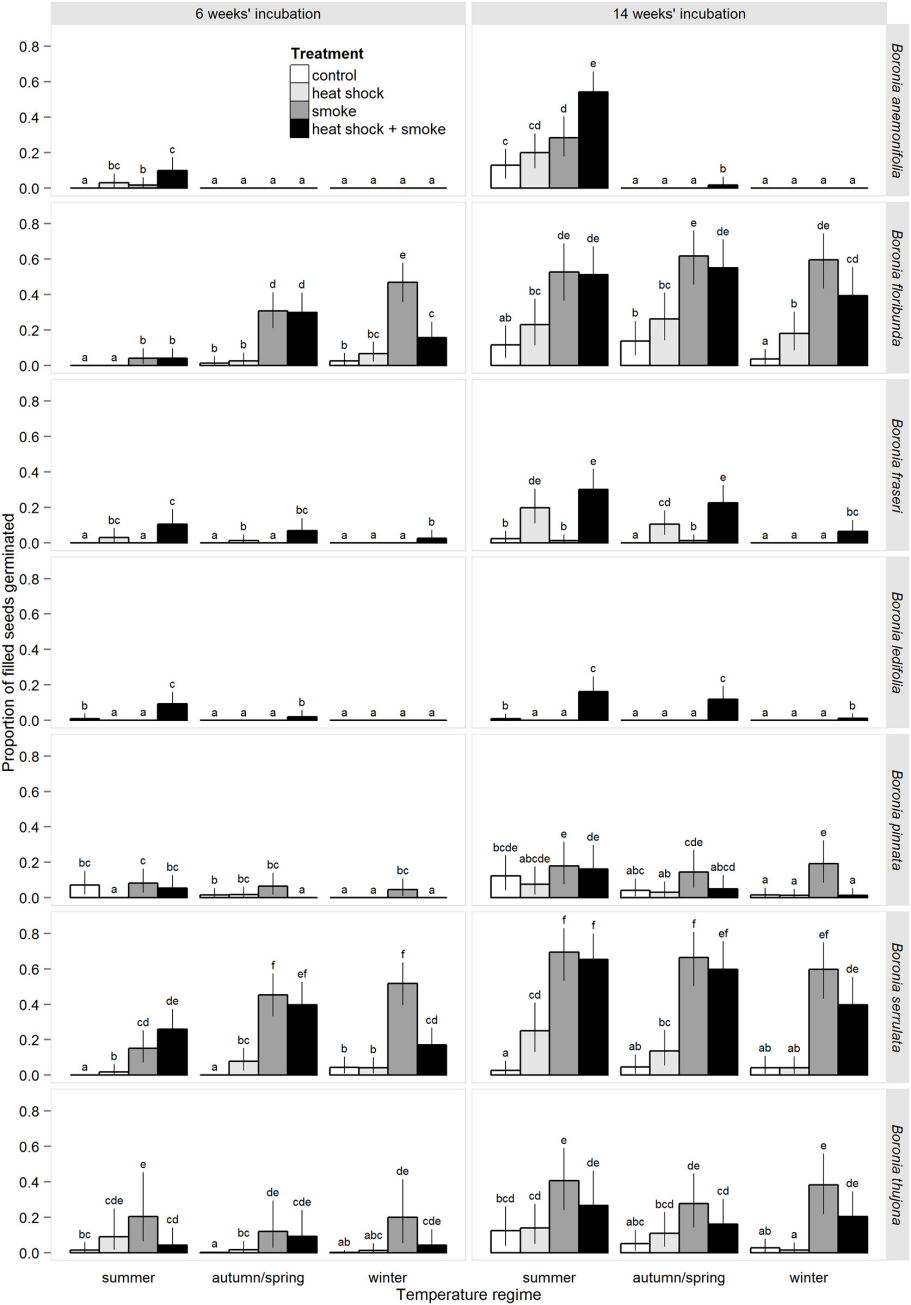
Predicted seed germination of seven species of *Boronia* from south eastern Australia in response to factorial combinations of heat shock, smoke and seasonal temperature regimes after 6 and 14 weeks’ incubation. Bar colours represent treatments as per the legend. Error bars are 95% Bayesian credible intervals. Different letters above bars indicate significant differences between means at α = 0.05.

Three broad patterns in seed morphology were recognisable amongst the study species and these corresponded with the three evolutionary lineages (‘sections’) represented: i) relatively small seeds with an elaiosome and dull, textured surfaces (1 spp., section Cyanothamnus); ii) relatively small seeds with smooth, shiny surfaces and no elaiosome (4 spp., section Boronia); and iii) much larger seeds with an elaiosome and shiny, textured surfaces (2 spp., section Valvatae) (Tables 1 and 3). Seed fill and viability of filled seeds were consistently high for all species (≥ 70% and ≥85%, respectively; Table 3). Maximum germination after 6 weeks’ incubation was low for most species (c. 10–20%), suggesting a high level of dormancy at the time of dispersal (c. 80–90%, Table 3). Notable exceptions were *B*. *floribunda* and *B*. *serrulata*, each with c. 50% of seeds non-dormant and able to germinate over a narrow range of conditions. Further incubation up to 14 weeks resulted in only moderate improvements to the maximum germination responses observed for all species after 6 weeks (*B*. *anemonifolia* was an exception with an increase of c. 40%; Fig 1). The extended incubation did, however, promote germination over a wider range of conditions for *B*. *anemonifolia*, *B*. *floribunda* and *B*. *serrulata* (Fig 1).

**Table 3:** Seed traits of seven species of *Boronia* from south eastern Australia. All seeds were black with linear, well-developed embryos. Quantitative data are means with standard errors in brackets.

Species	Lustre	Texture	Shape		Elaiosome	Seed weight		Seed fill		Viability of filled seeds	Primary dormancy
			lateral	ventral		(*n*)	(mg)	(*n*)	(%)	(%)	(%)
*B*. *anemonifolia*	dull	colliculate with hilly protrusions	ovoid	ovoid	present	57	1.39 (0.05)	79	78.5 (4.6)	93.5 (3.1)	90.0 (3.6)
*B*. *floribunda*	shiny	smooth	ellipsoid	ellipsoid	absent	80	1.28 (0.03)	79	89.9 (3.4)	95.8 (2.4)	53.2 (5.7)
*B*. *fraseri*	shiny	colliculate	ovoid	ovoid	present	80	6.88 (0.13)	81	97.5 (1.7)	98.7 (1.3)	89.4 (3.8)
*B*. *ledifolia*	shiny	colliculate	ovoid	ovoid	present	100	8.91 (0.19)	100	93.0 (2.6)	97.8 (1.5)	90.8 (3.1)
*B*. *pinnata*	shiny	smooth	ellipsoid	ellipsoid	absent	62	1.21 (0.05)	72	77.8 (4.9)	92.9 (3.4)	91.7 (3.6)
*B*. *serrulata*	shiny	smooth	ellipsoid	ellipsoid	absent	80	0.89 (0.03)	80	82.5 (4.2)	95.5 (2.6)	48.5 (6.2)
*B*. *thujona*	shiny	smooth	ellipsoid	ellipsoid	absent	80	2.02 (0.05)	72	87.5 (3.9)	98.4 (1.6)	79.6 (9.9)

### Effect of seasonal temperatures on germination

The range of seasonal temperatures required for germination varied between species and often interacted with heat shock and/or smoke (Table 4). Germination of *B*. *anemonifolia* was restricted almost entirely to summer temperatures (Fig 1). Summer and autumn/spring temperatures also promoted the greatest germination of *B*. *fraseri* and *B*. *ledifolia* (Fig 1), although overall responses for these two species were low. In contrast, winter and autumn/spring temperatures enhanced germination of *B*. *floribunda* early in the study, with no apparent effect at the end of the study (Fig 1). Similarly for *B*. *serrulata*, there was a positive effect of winter temperatures on earlier germination and this was mitigated by interactions with heat shock and smoke, respectively (Fig 1, Table 4). No effect of seasonal temperature was apparent at the end of the study except for a persistent negative interaction between heat shock and winter temperatures. Winter temperatures suppressed germination of *B*. *pinnata* (Fig 1) but this effect was mitigated by an interaction with smoke. No clear trends in germination with seasonal temperature were apparent for *B*. *thujona* (Fig 1) with the exception of a negative interaction between heat shock and winter temperatures (Table 4).

**Table 4:** Estimated posterior means, standard errors and 95% Bayesian credible intervals of regression coefficients for the combined effects of heat shock (H), smoke (S) and seasonal temperature treatments (autumn/spring regime, Se_Aut/Spr_; winter regime, Se_Win_) on seed germination of seven species of *Boronia* from south eastern Australia after 6 and 14 weeks’ incubation.

		6 weeks’ incubation					14 weeks’ incubation				
Species	Parameter	Mean	SD	2.5%	97.5%		Mean	SD	2.5%	97.5%	
*B*. *anemonifolia*	Intercept	-47.615	30.500	-118.896	-6.534	*	-1.974	0.405	-2.849	-1.259	*
	H	43.903	30.502	2.797	115.068	*	0.564	0.509	-0.413	1.583	ns
	S	42.965	30.526	1.760	114.597	*	1.034	0.499	0.111	2.078	*
	Se_Aut/Spr_	-89.318	69.321	-238.973	26.099	ns	-92.514	55.753	-219.659	-9.652	*
	Se_Win_	-92.062	69.114	-241.368	25.453	ns	-103.619	64.039	-245.734	-9.338	*
	H:S	-41.522	30.526	-113.120	-0.385	*	0.549	0.640	-0.728	1.770	ns
	H:Se_Aut/Spr_	-47.576	78.788	-211.733	99.482	ns	6.041	61.561	-115.821	130.520	ns
	H:Se_Win_	-45.203	81.504	-212.924	109.557	ns	-42.295	80.314	-207.549	108.417	ns
	S:Se_Aut/Spr_	-45.739	79.150	-212.142	102.713	ns	6.405	63.179	-120.308	131.496	ns
	S:Se_Win_	-48.558	79.967	-214.210	99.176	ns	-45.500	82.927	-218.745	106.231	ns
	H:S:Se_Aut/Spr_	-19.145	90.982	-198.711	152.910	ns	75.271	64.905	-43.883	209.998	ns
	H:S:Se_Win_	-14.235	90.914	-201.106	155.939	ns	-17.004	91.448	-201.971	158.717	ns
*B*. *floribunda*	Intercept	-42.026	25.552	-102.180	-6.439	*	-2.108	0.473	-3.086	-1.232	*
	H	-14.379	32.896	-84.400	46.678	ns	0.866	0.610	-0.284	2.098	ns
	S	38.719	25.553	3.175	98.665	*	2.222	0.581	1.110	3.416	*
	Se_Aut/Spr_	37.167	25.582	1.577	97.078	*	0.222	0.629	-1.012	1.468	ns
	Se_Win_	38.138	25.557	2.522	98.122	*	-1.364	0.829	-3.121	0.182	ns
	H:S	14.361	32.875	-46.592	84.465	ns	-0.933	0.780	-2.479	0.591	ns
	H:Se_Aut/Spr_	15.359	32.924	-45.575	85.294	ns	-0.038	0.816	-1.648	1.565	ns
	H:Se_Win_	15.533	32.893	-45.194	85.255	ns	1.052	0.994	-0.837	3.050	ns
	S:Se_Aut/Spr_	-34.685	25.583	-94.600	0.932	ns	0.154	0.788	-1.413	1.724	ns
	S:Se_Win_	-34.958	25.560	-95.100	0.642	ns	1.649	0.963	-0.176	3.633	ns
	H:S:Se_Aut/Spr_	-15.385	32.902	-85.457	45.679	ns	-0.176	1.065	-2.302	1.900	ns
	H:S:Se_Win_	-17.094	32.875	-86.902	43.614	ns	-1.828	1.217	-4.261	0.505	ns
	*σ*	-	-	-	-		0.447	0.164	0.100	0.771	
*B*. *fraseri*	Intercept	-81.039	46.652	-189.578	-10.766	*	-3.935	0.806	-5.800	-2.635	*
	H	77.292	46.674	7.092	185.989	*	2.514	0.869	1.024	4.450	*
	S	-36.326	57.079	-154.544	68.440	ns	-0.962	1.504	-4.242	1.743	ns
	Se_Aut/Spr_	-30.210	58.209	-151.234	78.083	ns	-40.346	30.010	-112.413	-2.271	*
	Se_Win_	-64.314	66.559	-203.673	55.616	ns	-91.551	55.179	-216.155	-7.581	*
	H:S	37.877	57.100	-66.645	155.921	ns	1.534	1.563	-1.294	4.918	ns
	H:Se_Aut/Spr_	29.045	58.241	-79.638	150.690	ns	39.566	30.008	1.559	111.707	*
	H:Se_Win_	-17.176	69.458	-156.544	120.939	ns	5.056	62.445	-120.815	128.291	ns
	S:Se_Aut/Spr_	-9.893	64.949	-138.883	116.376	ns	40.401	30.014	2.062	112.543	*
	S:Se_Win_	27.242	71.864	-113.053	168.404	ns	7.020	63.095	-120.363	132.376	ns
	H:S:Se_Aut/Spr_	10.570	64.913	-116.105	139.247	ns	-40.024	30.017	-112.164	-1.683	*
	H:S:Se_Win_	52.576	72.660	-83.948	198.599	ns	77.571	66.002	-46.261	212.856	ns
*B*. *ledifolia*	Intercept	-5.191	1.330	-8.440	-3.220	*	-5.189	1.340	-8.446	-3.224	*
	H	-53.764	40.739	-152.417	-1.916	*	-56.865	42.022	-157.670	-2.149	*
	S	-53.651	39.789	-148.969	-1.879	*	-56.123	41.478	-154.237	-1.927	*
	Se_Aut/Spr_	-80.290	54.563	-208.485	-4.076	*	-79.050	54.039	-203.901	-3.891	*
	Se_Win_	-94.228	63.259	-236.932	-4.746	*	-80.418	55.504	-209.685	-4.099	*
	H:S	110.264	51.437	25.761	223.400	*	116.499	53.125	28.970	234.683	*
	H:Se_Aut/Spr_	13.837	70.316	-126.376	150.688	ns	17.370	70.196	-127.550	151.992	ns
	H:Se_Win_	-35.606	84.355	-210.108	120.263	ns	13.070	70.799	-128.349	153.490	ns
	S:Se_Aut/Spr_	14.504	71.097	-127.713	152.230	ns	15.111	71.869	-130.441	156.260	ns
	S:Se_Win_	-38.279	86.363	-213.845	121.769	ns	14.270	70.865	-129.775	150.960	ns
	H:S:Se_Aut/Spr_	50.161	73.731	-85.492	198.530	ns	46.205	74.235	-94.158	198.171	ns
	H:S:Se_Win_	-25.017	91.302	-209.995	148.710	ns	49.605	75.375	-87.837	204.094	ns
*B*. *pinnata*	Intercept	-2.680	0.546	-3.855	-1.727	*	-2.052	0.488	-3.084	-1.160	*
	H	-36.558	27.712	-105.916	-2.724	*	-0.580	0.783	-2.173	0.916	ns
	S	0.187	0.735	-1.241	1.631	ns	0.470	0.648	-0.776	1.763	ns
	Se_Aut/Spr_	-2.052	1.428	-5.374	0.221	ns	-1.264	0.862	-3.071	0.340	ns
	Se_Win_	-59.207	42.275	-159.294	-4.541	*	-2.621	1.290	-5.561	-0.445	*
	H:S	36.041	27.725	2.298	105.569	*	0.456	1.004	-1.500	2.468	ns
	H:Se_Aut/Spr_	36.691	27.780	2.542	105.489	*	0.157	1.349	-2.508	2.809	ns
	H:Se_Win_	-67.432	76.461	-230.744	67.021	ns	0.255	2.028	-4.018	4.270	ns
	S:Se_Aut/Spr_	1.769	1.592	-0.961	5.291	ns	0.997	1.061	-1.031	3.123	ns
	S:Se_Win_	58.518	42.263	3.861	158.486	*	2.711	1.416	0.196	5.896	*
	H:S:Se_Aut/Spr_	-106.479	58.282	-243.549	-19.763	*	-1.310	1.702	-4.717	2.019	ns
	H:S:Se_Win_	-49.466	81.939	-214.847	108.512	ns	-3.453	2.446	-8.188	1.534	ns
	*σ*	-	-	-	-		0.408	0.274	0.020	1.029	
*B*. *serrulata*	Intercept	-37.022	22.383	-89.009	-6.280	*	-3.875	0.871	-5.817	-2.424	*
	H	32.404	22.394	1.555	84.253	*	2.749	0.954	1.095	4.801	*
	S	35.257	22.388	4.520	87.158	*	4.719	0.942	3.091	6.747	*
	Se_Aut/Spr_	-22.179	37.312	-103.646	42.096	ns	0.659	1.133	-1.525	2.976	ns
	Se_Win_	33.768	22.385	3.001	85.547	*	0.568	1.106	-1.527	2.854	ns
	H:S	-31.713	22.399	-83.349	-0.848	*	-2.943	1.069	-5.203	-0.974	*
	H:Se_Aut/Spr_	24.229	37.302	-40.022	106.006	ns	-1.450	1.287	-4.024	1.041	ns
	H:Se_Win_	-32.439	22.401	-84.364	-1.583	*	-2.792	1.353	-5.599	-0.226	*
	S:Se_Aut/Spr_	23.747	37.319	-40.552	105.242	ns	-0.800	1.245	-3.305	1.609	ns
	S:Se_Win_	-31.935	22.393	-83.902	-1.121	*	-1.005	1.211	-3.439	1.300	ns
	H:S:Se_Aut/Spr_	-25.147	37.305	-107.004	39.153	ns	1.346	1.469	-1.505	4.273	ns
	H:S:Se_Win_	30.070	22.409	-0.945	82.010	ns	2.148	1.523	-0.771	5.293	ns
	*σ*	-	-	-	-		0.426	0.192	0.063	0.818	
*B*. *thujona*	Intercept	-5.050	1.395	-8.223	-2.779	*	-3.875	0.871	-5.817	-2.424	*
	H	2.504	1.535	-0.108	5.841	ns	2.749	0.954	1.095	4.801	*
	S	3.583	1.461	1.143	6.827	*	4.719	0.942	3.091	6.747	*
	Se_Aut/Spr_	-4.038	2.075	-8.501	-0.313	*	0.659	1.133	-1.525	2.976	ns
	Se_Win_	-2.764	2.066	-7.380	0.801	ns	0.568	1.106	-1.527	2.854	ns
	H:S	-4.600	1.734	-8.310	-1.614	*	-2.943	1.069	-5.203	-0.974	*
	H:Se_Aut/Spr_	1.754	1.505	-0.999	4.955	ns	-1.450	1.287	-4.024	1.041	ns
	H:Se_Win_	0.188	1.494	-2.645	3.279	ns	-2.792	1.353	-5.599	-0.226	*
	S:Se_Aut/Spr_	3.339	1.919	-0.142	7.411	ns	-0.800	1.245	-3.305	1.609	ns
	S:Se_Win_	2.722	1.925	-0.713	6.939	ns	-1.005	1.211	-3.439	1.300	ns
	H:S:Se_Aut/Spr_	-	-	-	-		1.346	1.469	-1.505	4.273	ns
	H:S:Se_Win_	-	-	-	-		2.148	1.523	-0.771	5.293	ns
	*σ*	0.828	0.403	0.114	1.731		0.426	0.192	0.063	0.818	

* indicates significant at α = 0.05;

ns, non-significant.

### Germination response to fire cues

Germination of freshly dispersed seeds was tightly cued to fire, with negligible germination in the absence of fire-related germination cues (Fig 1). The combined effects of heat shock and smoke varied with species, seasonal temperatures and duration of incubation from neutral to additive, synergistic, unitive or negative, and could not be reliably predicted from their effects when applied in isolation. Smoke had a significant positive effect on the germination of all seven species, either as a solitary cue in *B*. *anemonifolia*, *B*. *floribunda*, *B*. *pinnata*, *B*. *serrulata* and *B*.*thujona*, or in combination with heat shock in *B*. *fraseri* and *B*. *ledifolia* (Fig 1, Table 4). The maximum germination response observed for each species was invariably associated with treatments involving smoke (smoke only or heat shock plus smoke), although for *B*. *fraseri* and *B*. *pinnata* this response was not significantly different to one or more non-smoke treatments (Fig 1).

Heat shock had varied effects on germination depending on the species, seasonal temperatures, duration of incubation and the presence or absence of smoke (Fig 1, Table 4). Its effect as an isolated cue on germination of *B*. *anemonifolia* was negligible but the combined effect of heat shock and smoke was synergistic after 6 weeks and additive after 14 weeks, resulting in the maximum germination response (Fig 1, Table 4). Heat shock had a consistently positive effect on *B*. *fraseri* (Fig 1) with evidence for a significant heat shock × smoke × autumn/spring temperatures interaction after 14 weeks’ incubation (Table 4). A unitive effect of heat shock and smoke in *B*. *ledifolia* meant zero germination in the absence of either cue (Fig 1). Heat shock promoted germination of *B*. *serrulata* as an isolated cue but a negative heat shock × winter temperature interaction reduced the efficacy of smoke when the two cues were combined at winter temperatures (Table 4, Fig 1). The combined effect of heat shock and smoke was also negative in *B*. *thujona* with heat shock diminishing the effectiveness of smoke (Fig 1). Heat shock effects on germination after 6 weeks’ incubation in *B*. *pinnata* were no longer apparent at the conclusion of the study (Table 4), and the earlier germination responses are considered too negligible to be reliable. The heat shock treatment increased seed mortality in several of the smaller-seeded species. Reductions in mean post-trial viability relative to the control treatments ranged from 15–29% for *B*. *floribunda*, 32–43% for *B*. *pinnata*, and 24–35% for *B*. *thujona*, while the other study species were much less affected.

### Temporal patterns in germination

**Fig 2:**
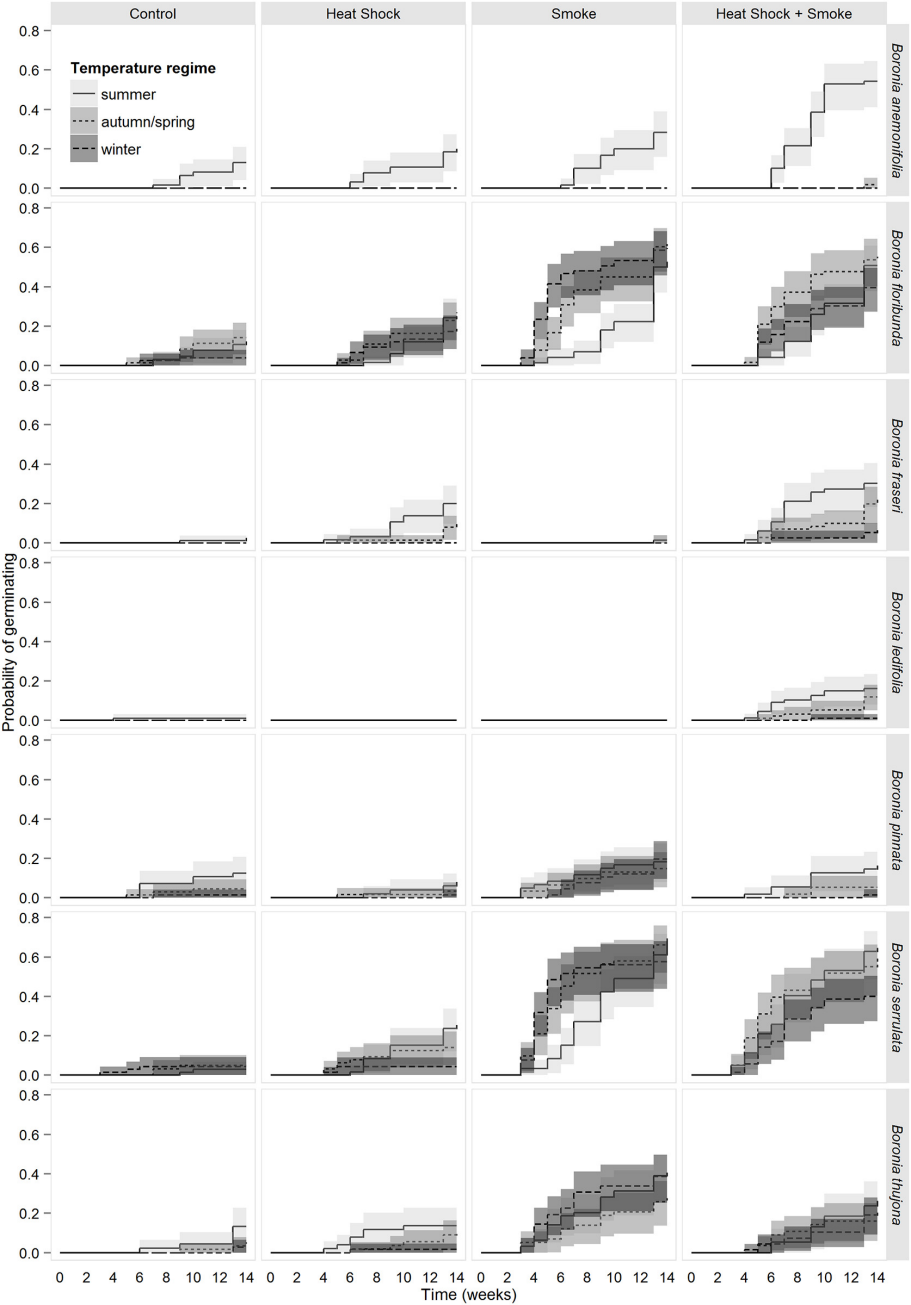
Kaplan-Meier estimates of survivor functions for seeds of seven species of *Boronia* from south eastern Australia treated with factorial combinations of heat shock, smoke and seasonal temperature regimes. Line types represent seasonal temperature regimes as per the legend. Shading indicates point-wise 95% confidence intervals.

The probability of germination expressed through survivor functions showed complex interactions between seasonal temperature and fire cues (Fig 2). The minimum time to onset of germination ranged from three to six weeks depending on the species. To facilitate comparisons of relative germination rates between treatments and species, many of which had large proportions of ungerminated seeds at the conclusion of the study, we examined the time to 20% germination and compared this with the time to onset of germination (Table 5).

**Table 5:** Timing of germination for seven species of *Boronia* from south eastern Australia in response to heat shock, smoke and seasonal temperatures, as estimated by non-parametric Kaplan-Meier survivor functions. Prior to incubation seeds received no treatment (control, (C)), heat shock (H), smoke (S), or heat shock plus smoke (H+S). Relative germination rates were not derived for treatments with less than 20% mean final germination.

Species	Daily maxima / minima (°C)	Time to onset of germination (t_0_) (weeks)				Time to 20% germination (t_20_) (weeks)				Relative germination rate (1/(t_20_-t_0_)			
		C	H	S	H+S	C	H	S	H+S	C	H	S	H+S
*B*. *anemonifolia*	22/12	7	6	6	6	-	14	10	7	-	0.13	0.25	1.00
	17/6	-	-	-	13	-	-	-	-	-	-	-	-
	10/3	-	-	-	-	-	-	-	-	-	-	-	-
*B*. *floribunda*	26/17	7	7	4	5	-	13	10	9	-	0.17	0.17	0.25
	22/12	5	5	4	4	-	13	6	5	-	0.13	0.50	1.00
	17/6	6	5	3	5	-	-	4	7	-	-	1.00	0.50
*B*. *fraseri*	26/17	9	4	13	4	-	13	-	7	-	0.11	-	0.33
	22/12	-	5	13	5	-	-	-	14	-	-	-	0.11
	17/6	-	-	-	6	-	-	-	-	-	-	-	-
*B*. *ledifolia*	26/17	4	-	-	4	-	-	-	-	-	-	-	-
	22/12	-	-	-	5	-	-	-	-	-	-	-	-
	17/6	-	-	-	9	-	-	-	-	-	-	-	-
*B*. *pinnata*	22/12	6	7	3	4	-	-	-	-	-	-	-	-
	17/6	5	5	3	7	-	-	-	-	-	-	-	-
	10/3	7	13	5	13	-	-	-	-	-	-	-	-
*B*. *serrulata*	26/17	9	6	3	3	-	13	7	5	-	0.14	0.25	0.50
	22/12	7	4	3	3	-	-	4	5	-	-	1.00	0.50
	17/6	3	4	3	3	-	-	4	7	-	-	1.00	0.25
*B*. *thujona*	26/17	6	4	3	6	-	-	7	13	-	-	0.25	0.14
	22/12	9	6	3	5	-	-	10	-	-	-	0.14	-
	17/6	13	6	3	4	-	-	6	14	-	-	0.33	0.10

Time to onset for *B*. *anemonifolia* at summer temperatures was unaffected by treatment, however, subsequent germination was fastest in response to a combination of heat shock and smoke, followed by smoke only, and heat shock only (Fig 2, Table 5). Smoke promoted the fastest onset of germination in *B*. *floribunda*, *B*. *pinnata*, *B*. *serrulata* and *B*. *thujona*, and across all seasonal temperatures examined (Fig 2, Table 5), however the combined heat shock plus smoke treatment was just as effective for *B*. *floribunda* at autumn/spring temperatures, and for *B*. *serrulata* across all temperatures. Subsequent germination of *B*. *floribunda* was fastest in the presence of smoke-related treatments (i.e. smoke only or heat shock plus smoke), and occurred up to six times faster at winter and autumn/spring temperatures than at summer temperatures. Germination of *B*. *serrulata* in response to smoke treatment was four times faster at cooler temperatures (autumn/spring and winter) than at summer temperatures, and two to four times faster than the combined heat shock plus smoke treatment at cooler temperatures (autumn/spring and winter). Smoke treatment also resulted in two to three times faster germination of *B*. *thujona* relative to the combined heat shock and smoke treatment with no consistent effect of seasonal temperature on germination speed. Germination of *B*. *ledifolia* and *B*. *pinnata* was insufficient to discern patterns in relative germination rates between treatments although winter temperatures consistently delayed the onset of germination relative to warmer temperatures. The time to onset of germination of *B*. *fraseri* was the same in response to heat shock and the heat shock plus smoke treatment (the only treatments to produce substantial germination), however, germination commenced one to two weeks earlier at summer temperatures. The combined heat shock plus smoke treatment promoted three times faster germination at summer temperatures compared with autumn/spring temperatures, and in comparison with the heat shock only treatment at summer temperatures.

## Discussion

### Seasonal germination requirements

Seasonal temperatures influenced the timing and magnitude of germination in response to fire cues for the majority of species examined. This highlights the role of seasonal temperatures—not just direct fire cues such as heat shock and smoke—as important drivers of germination in fire-prone regions [15]. In Mediterranean-type climates, post-fire germination is highly seasonal and coincides with reliable winter rains. Hence, fire season has the potential to cause significant delays in post-fire seedling emergence as the timing of fire can vary while germination is confined to the wetter months [13,17]. In contrast, in non-Mediterranean climates such as fire-prone south eastern Australia, rainfall is aseasonal and more variable between years. Our results suggest that post-fire seedling emergence does not necessarily occur as soon as adequate soil moisture is available. Rather, germination depends on ambient temperature, and hence will be affected by the season of fire occurrence. This corroborates relatively recent evidence of seasonal emergence patterns in the region [18], and that fires in certain seasons can cause significant delays in the timing of seedling emergence of such species with negative repercussions for subsequent recruitment success [9].

Our results suggest two mechanisms by which *Boronia* and other genera with PD in fire-prone regions might be expected to exhibit seasonal emergence patterns. The first is where germination is restricted to a particular seasonal temperature range and simply does not occur outside that range (a seasonal *requirement*). This mechanism is best exemplified by *B*. *anemonifolia* where germination of fresh seeds only occurred at summer temperatures (Fig 1). The second mechanism that is expected to lead to seasonal emergence patterns is temperature-dependent rates of germination, whereby species may be capable of germinating across a range of seasonal temperatures but exhibit much faster and/or greater germination at certain temperatures than others (a seasonal *preference*). The window within which soils remain moist enough to facilitate seedling emergence and establishment after a given rainfall event is likely to narrow as temperature increases, and soils are expected to dry out much faster in summer than in autumn/spring or winter [78]. For *B*. *floribunda* and *B*. *serrulata*, comparable levels of maximum germination had been reached across all three seasonal temperatures by the end of the experiment (Fig 1), however, the additional time required to reach these levels at summer temperatures (Fig 2, Table 5) is likely to confine the majority of seedling emergence to the cooler months of the year. Provided rainfall is adequate, this suggests that fires occurring in late-spring to summer, the historical or pre-European peak fire season in the region [54], could delay seedling emergence until the following autumn, while fires occurring in late autumn to winter, the peak time for prescribed management fires, would result in rapid post-fire germination with earlier exposure of germinants to desiccating conditions and higher risk of mortality over the following summer. The reverse pattern is true for *B*. *fraseri* and *B*. *ledifolia*, the seeds of which germinated faster and in greater numbers at summer temperatures than at autumn/spring temperatures, and for which germination was negligible at winter temperatures (Figs 1 and 2). This suggests that seedling emergence in these species will be largely confined to the warmer part of the year, implying faster post-fire seedling emergence following spring to early summer fires and substantial delays caused by fires occurring in late summer to autumn. The larger seed size of these species (Table 3) might also be an adaptive response to selective pressure of summer drought on newly emerged seedlings. Further work comparing seed lots from different provenances throughout each species’ range is needed to determine the degree of plasticity in the seasonal temperatures required for germination, and how this affects germination phenology and recruitment success at different sites [79,80].

### Effect of fire cues on germination

Germination of freshly dispersed seeds was negligible in the absence of fire cues, reflecting the important role that fires play in regulating seedling recruitment in wild populations of these species. A germination requirement for one or more fire-related stimuli ensures that the bulk of the annual seed crop will enter the soil seed bank and remain available, subject to sufficient seed longevity and escape from predation, for population recovery in the event of fire. In this way, depletion of valuable seed reserves through germination is restricted to the immediate post-fire window where competition for resources such as light, space and nutrients is lowest, and the chances of successful seedling establishment are greatest. This is especially pertinent for obligate-seeding species (all of the study species except *B*. *pinnata*) whose above-ground populations may be completely eliminated by a single fire event (however, see Ooi *et al*. [81]).

The maximum germination response observed for each species was in response to some form of smoke treatment, either as a solitary cue (5 spp.) or in combination with heat shock (2 spp.) (Fig 1), further emphasising the strong relationship between PD and smoke-promoted germination. This finding is consistent with positive responses to smoke only treatments reported for Rutaceae in Western Australia [32,82] and south eastern Australia [58]. Karrikins [83,84] have been identified as the main bioactive agents in smoke that stimulate germination, along with cyanohydrins [85] and potentially nitrate and nitrogen oxides [35,86], and their mode of action is an area of active research [35]. The temperature-dependent effects of the smoke response observed for several species in the present study highlight the importance of examining a full complement of seasonal temperatures in conjunction with the smoke cue in studies of PD species. Failure to do so could increase the likelihood of false-negative conclusions [35] and preclude identification of important interactions between smoke and ambient temperature.

The promotive effect of heat shock on the germination of hard-seeded (physically dormant) species is widely known, however, there are relatively few reports of positive responses to heat shock in species with PD e.g. some Apiaceae [14], Ericaceae [29,37], Haemodoraceae [24,45], Lamiaceae [29], Myrtaceae [68], Poaceae [41], Proteaceae [39] and Rutaceae [57,69,87]. In the present study, we found that heat shock was essential for germination of *B*. *fraseri* and *B*. *ledifolia*, and that it enhanced the speed and magnitude of germination of *B*. *anemonifolia* in the presence of smoke (Fig 2). As an isolated cue, heat shock had little effect on the magnitude of germination of the remaining study species, however, both positive and adverse effects of heat shock on the speed of germination in response to smoke were observed in *B*. *floribunda* and *B*. *serrulata*, and these effects varied with seasonal temperature (Fig 2). The degree and duration of soil heating during the passage of fires is, therefore, expected to influence post-fire recruitment in at least some *Boronia* spp., and possibly in many other genera with PD. This is a well-recognised phenomenon in the physically dormant species that dominate many fire-prone ecosystems [27,30,31,67,88] and this study highlights its importance for species with other dormancy types. A heat shock requirement for, or effect on, germination has important implications for fire management for conservation, particularly in relation to fire intensity. High intensity fires produce deeper penetration of heat shock cues into the soil profile due to greater consumption of fine fuels at the soil surface [67]. Prescribed fires, however, tend to be lower in intensity and to burn patchily; hence, they may generate insufficient soil heating to promote germination of many soil-stored seeds [67,88]. Further research is needed to identify the range of temperatures and durations of heating that are optimal for germination of heat-responsive *Boronia* spp., as well as those that are lethal to seeds, and how these are influenced by other fire and environmental cues. Characterising the response surfaces to heat shock for a greater range of plant families and functional types would greatly inform fire management practices for conservation [27].

In physically dormant species, heat shock is a dormancy-breaking mechanism whereby exposure to extreme temperatures breaks an impermeable seed coat which otherwise prevents imbibition and germination. In PD species with permeable seed coats, however, the mechanism by which heat shock promotes germination is unknown. For species with larger seeds and thicker testas, such as *B*. *fraseri* and *B*. *ledifolia*, it may be that the testa is restricting radicle growth and embryo enlargement, and that heat shock weakens the structural integrity of the testa and lowers its resistance to radicle penetration. Reports of positive responses to nicking or removing sections of testa in other Australian Rutaceae are consistent with this interpretation [89–92].

### Overcoming dormancy

The limited germination responses observed for several species are attributable to high levels of primary dormancy which was not overcome during the study, rather than to application of inappropriate germination cues. These species require some form of seasonal stratification, after-ripening and/or other biological process during burial before the majority of seeds will lose dormancy and their responsiveness to germination cues such as heat shock, smoke and seasonal temperatures can be adequately determined [16,60,73]. Treatment with gibberellic acid, a phytohormone known to promote germination of a large number of physiologically dormant species [93], has had mixed results in laboratory trials using Australian Rutaceae [58]. Further research using buried seeds in natural populations is needed to establish when and how dormancy is overcome and how it changes with time, and to better understand the combinations of cues that promote germination once seeds are fully non-dormant. Notwithstanding this, the germination syndromes observed in this study have highlighted some important implications for the persistence of PD species in fire-prone floras under a changing climate.

### Functional classification of germination syndromes

Germination responses were highly variable between the study species even though they are closely related congeners with comparable habitat requirements, including several taxa that co-occur. Aside from *B*. *pinnata*, where overall responses were too low to elucidate its germination requirements, the range of observed responses could be broadly characterised by three main germination syndromes: 1) germination is greatest in response to a combination of heat shock and smoke, and is entirely restricted to summer temperatures (*B*. *anemonifolia*); 2) germination is greatest in response to smoke and either a) cooler seasonal temperatures promote faster germination (*B*. *floribunda* and *B*. *serrulata*), or b) there is no apparent effect of seasonal temperatures (*B*. *thujona*); and 3) heat shock is essential for germination and warmer seasonal temperatures promote the greatest and fastest responses (*B*. *fraseri* and *B*. *ledifolia*). Notably, these species groupings/syndromes are consistent with the three broad patterns in seed morphology identified in the study species (Table 3), which correspond, in turn, with the three separate evolutionary lineages represented: namely, section Cyanothamnus (syndrome 1 species), section Boronia (syndrome 2 species plus *B*. *pinnata*); and section Valvatae (syndrome 3 species). Hence, patterns in seed morphology and/or interspecific phylogenetic relationships could be useful predictors of germination syndromes in hitherto unstudied members of the genus and family, as well as in other plant families with poorly known or highly variable germination requirements. Recognising such patterns and relationships between candidate taxa *a priori*, and explicitly incorporating them into the design of future comparative studies of seed ecology, could vastly improve our ability to identify important functional groups based on shared responses [21], and provide a strong foundation for extrapolation of results and prediction of species’ and ecosystem responses to environmental change.

### Conclusions

The evidence for seasonal germination requirements in the Rutaceae presented in this study, together with reports of seasonal emergence patterns in wide variety of PD taxa from fire-prone regions around the world including south eastern Australia [18], south western Australia [17], Florida [94], and the Cape Floristic Region of South Africa [13], suggests that seasonal germination requirements are a very widespread phenomenon in fire-prone floras, irrespective of differences in climate. Prescribed burning outside the peak wildfire season and climate change impacts on ambient temperatures and fire regimes are, therefore, likely to have both localised and widespread effects on the timing and magnitude of seedling recruitment in fire-prone ecosystems worldwide. Further research is needed to characterise the germination syndromes of a wider range of taxonomic and functional groups from a variety of fire-prone ecosystems in order to determine the full range of species potentially at risk. Imperative to this are future studies addressing the combined effects of multiple fire-related cues in conjunction with a full complement of seasonal ambient temperatures in order to provide valuable, ecologically meaningful insights into germination strategies and the mechanisms that govern recruitment phenology of PD species in fire-prone ecosystems.

Current fire management practices for biodiversity conservation are predicated almost exclusively on critical fire-return intervals and the avoidance of too frequent or infrequent fire for sensitive species [95]. This study contributes to a growing body of research on fire season effects and calls for an expansion of the current framework beyond fire frequency to address the additional impacts of fire intensity and fire season on native flora. This is especially pertinent given the projected widening of fire season and increasing intensity of wildfires as a result of climate change.

## Supporting Information

S1 Dataset An Excel file containing the raw germination data. (XLSX)
